# Vitamin D and Its Receptors in Cervical Cancer

**DOI:** 10.7150/jca.87499

**Published:** 2024-01-01

**Authors:** Hanyu Dong, Shiyue Chen, Xiaoshan Liang, Qiliang Cai, Xumei Zhang, Juan Xie, Zhuoyu Sun

**Affiliations:** 1 Department of Epidemiology and Biostatistics, School of Public Health, Tianjin Medical University, Tianjin, China.; 2 Tianjin Key Laboratory of Environment, Nutrition and Public Health, Tianjin, China.; 3 Tianjin Center for International Collaborative Research in Environment, Nutrition and Public Health, Tianjin, China.; 4 Department of Nutrition and Food Science, School of Public Health, Tianjin Medical University, Tianjin, China.; 5 Department of Urology, The Second Hospital of Tianjin Medical University, Tianjin, China.

**Keywords:** vitamin D, vitamin D receptor, cervical cancer

## Abstract

Several studies have investigated the relationship between vitamin D (VD) and its receptors (VDR) and the risk of cervical cancer. However, the underlying mechanisms that underpin these associations remain incompletely comprehended. In this review, we analyzed the impacts of VD and VDR on cervical cancer and related mechanisms, and discussed the effects of VD, calcium, and other vitamins on cervical cancer. Our literature research found that VD, VDR and their related signaling pathways played indispensable roles in the occurrence and progression of cervical cancer. Epidemiological studies have established associations between VD, VDR, and cervical cancer susceptibility. Current studies have shown that the inhibitory effect of VD and VDR on cervical cancer may be attributed to a variety of molecules and pathways, such as the EAG potassium channel, HCCR-1, estrogen and its receptor, p53, pRb, TNF-α, the PI3K/Akt pathway, and the Wnt/β-catenin pathway. This review also briefly discussed the association between VDR gene polymorphisms and cervical cancer, albeit a comprehensive elucidation of this relationship remains an ongoing research endeavor. Additionally, the potential ramifications of VD, calcium, and other vitamins on cervical cancer has been elucidated, yet further exploration into the precise mechanistic underpinnings of these potential effects is warranted. Therefore, we suggest that further studies should focus on explorations into the intricate interplay among diverse molecular pathways and entities, elucidation of the mechanistic underpinnings of VDR polymorphic loci changes in the context of HPV infection and VD, inquiries into the mechanisms of VD in conjunction with calcium and other vitamins, as well as investigations of the efficacy of VD supplementation or VDR agonists as part of cervical cancer treatment strategies in the clinical trials.

## Introduction

Cervical cancer has been identified as the fourth most common cancer and the fourth leading cause of cancer-related deaths among women worldwide, with approximately 600,000 new cases and 340,000 deaths reported annually 1. The pathogenesis of cervical cancer is a complicated biological process, which involves multi-stage, long-term progression, and multi-factor interactions. Cervical cancer typically originates from healthy cervical tissue and progresses through cervical intraepithelial neoplasia (CIN 1/2/3) to invasive cervical cancer 2, 3. Although the etiology of cervical cancer remains elusive, high-risk human papillomavirus (HR-HPV) is a significant risk factor, with subtypes 16 and 18 being the most prevalent. Despite the widespread occurrence of human papillomavirus (HPV) infections, more than 90% of women can clear the HPV infection within three years through their immune system after infection. Only 10% of patients may experience persistent HPV infection, and ultimately, less than 1% of those with persistent HPV infection will develop cervical cancer 4. The reasons for carcinogenesis are possibly related to environmental factors, including dietary patterns and lifestyle choices. Consequently, exploring the factors that lead to persistent HPV infection and ultimately result in the development of cervical cancer has been a prominent research focus in recent years.

The global prevalence of vitamin D deficiency has emerged as a significant health concern, which has raised concerns regarding its potential implications for persistent HPV infection and cervical cancer occurrence. The prevalence of vitamin D (VD) deficiency ranges from 6.9% to 81.8% in European nations and from 2.0% to 87.5% in Asian nations. In more than half the countries, VD deficiency is present in more than 50% of adult individuals 5. The presence of vitamin D receptors (VDR) and VD-activating enzymes in immune cells, such as monocytes, macrophages, dendritic cells, and lymphocytes, suggests that VD may act as an immunomodulator by binding VDR 6. Previous research indicated that women with compromised immunity were at elevated risk of harboring persistent HPV infection, which may subsequently progress to CIN and ultimately develop into cervical cancer 2, 3.

It is well known that VD can facilitate bone formation and calcification by stimulating the absorption of calcium and phosphorus by intestinal mucosal cells 7, 8. Notably, there is a growing body of evidence suggesting that VD and its metabolites may exert a significant role in the prevention or treatment of gynecological cancers, representing a novel and distinct function of VD that diverges from its established function in the regulation of calcium and bone metabolism 9. However, the precise anticancer mechanism of VD in cervical cancer is still unclear and requires further exploration. This study was proposed to elaborate on previous research regarding VD and VDR and cervical cancer, and to discuss the function and the underlying mechanisms of VD and VDR in cervical cancer.

## Metabolic and biological functions of VD

VD is a crucial nutrient required for maintaining various daily activities. It serves as a potent precursor for the steroid hormone that mainly regulates the balance of calcium and phosphorus in the human body and contributes to bone mineralization. In recent years, numerous studies have linked VD deficiency to several extra-skeletal diseases, including cancer, high blood pressure, and autoimmune diseases 10.

VD can be obtained from two different ways: body synthesis and dietary intake. It is pertinent to note that dietary sources of VD include fatty fish (e.g. salmon: 441IU per 100g), fortified foods such as fortified milk (40IU per 100g) and fortified cheese (301IU per 100g), and to a lesser extent, egg yolks (218IU per 100g) and “sun-dried” mushrooms (154IU per 100g) 11. However, dietary sources of VD alone are insufficient to meet the body's needs, making the primary source of VD synthesis in the skin via photochemistry in response to ultraviolet B (UVB) radiation.

**Figure 1:**
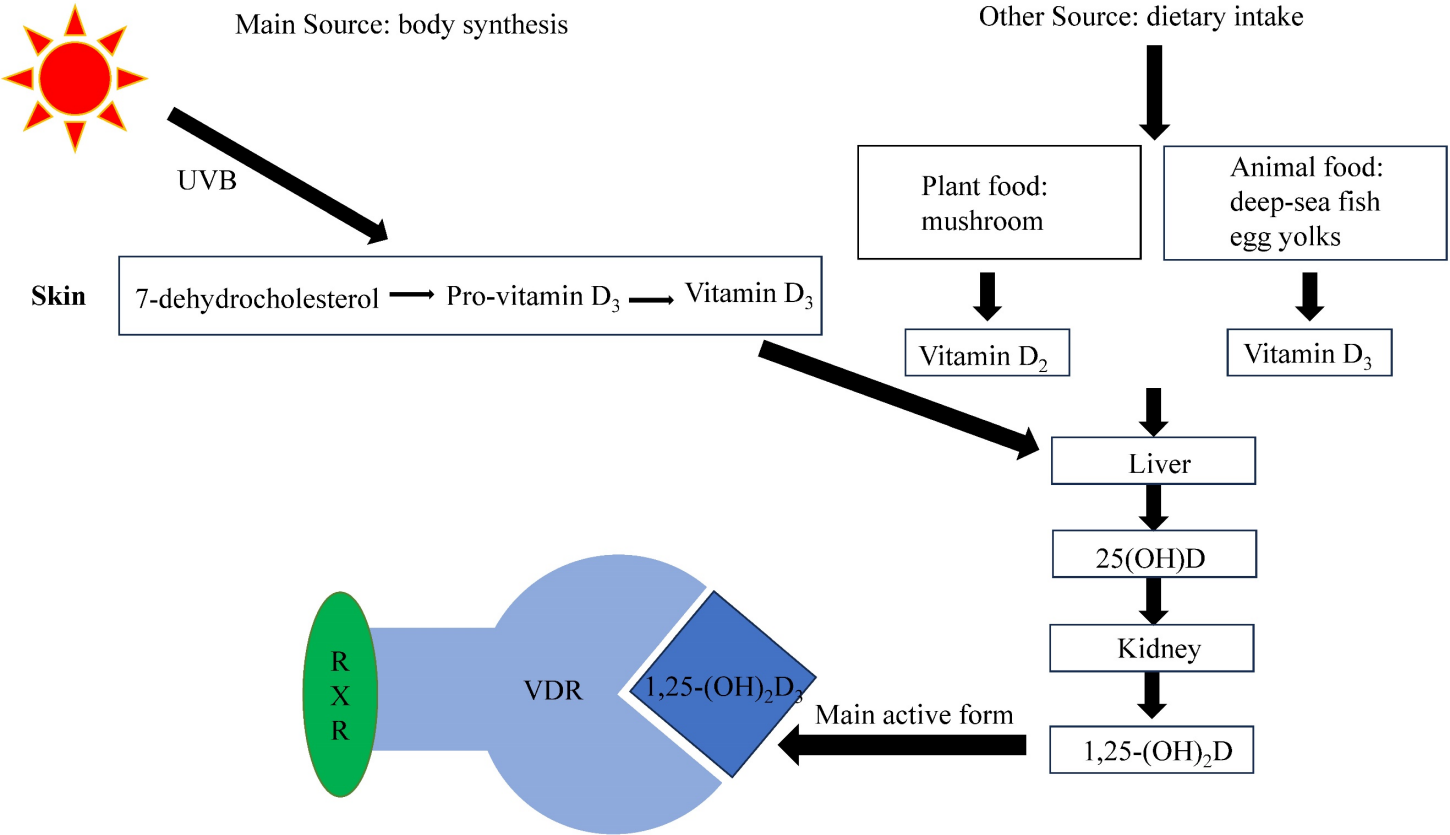
Synthesis and metabolism of VD^48^. VD is mostly produced by the skin under the influence of UVB radiation, although it can also be acquired through dietary sources. Upon circulation in the bloodstream, VD undergoes hepatic conversion into 25(OH)D. Subsequently, in the renal tissues, it undergoes further transformation into 1,25-(OH)_2_D, with 1,25-(OH)_2_D_3_ being the most biologically active form. To regulate gene expression, 1,25-(OH)_2_D_3_ interacts with VDR and forms complexes with RXR.

Currently, several types of VD exist, with VD_2_ and VD_3_ being the more important. VD_2_ (ergocalciferol) is mainly produced in plants, while VD_3_ (cholecalciferol), synthesized as a prohormone in the skin, is the body's primary source of VD 12. However, VD itself is not biologically active and needs to undergo two hydroxylation processes to convert into its biologically active form, 1,25-dihydroxyvitamin D 13. The liver converts VD into 25-hydroxyvitamin D (25(OH)D), which is the main type of VD in the body, and has a long half-life and a high serum concentration. The serum 25(OH)D level serves as an effective indicator of the body's VD levels. The kidneys then convert 25(OH)D into the active metabolite 1,25-dihydroxyvitamin D_,_ of which 1, 25-dihydroxyvitamin D_3_(1,25-(OH)_2_D_3_, calcitriol) is the most active, which exerts its physiological effects by binding to VD receptors (VDRs) (Figure 1).

Both 25(OH)D and 1,25-(OH)_2_D_3_ bind to VD binding protein and are transported in the blood. As 1,25-(OH)_2_D_3_ has a 1000-fold higher affinity for VDR than 25(OH)D, it is considered the primary effector of VDR binding 14, 15. The active metabolite 1,25-(OH)_2_D_3_ binds to VDR and regulates various physiological processes in the body 16, 17.

## The structure and biological function of VDR

VDR, as a transcription factor, reacts to 1,25-(OH)_2_D_3_ and mediates its biological effects, such as calcium homeostasis and immune response 18, 19. When activated by 1,25-(OH)_2_D_3_, VDR promotes the expression of genes responsible for enhancing calcium absorption from dietary sources into the bloodstream 18. Activated VDR can influence the immune system by modulating the production of cytokines, such as interleukin-2 (IL-2), and regulating the differentiation and function of immune cells 19. VDR can heterodimerize with retinoid X receptor (RXR) to bind to response elements on target genes and thereby modulate gene expression 20 (Figure 1). The VDR gene is located in chromosome 12, with hundreds of single nucleotide polymorphisms. Promoter methylation can cause epigenetic changes, but its effect on VDR expression remains controversial 21. The occurrence of VDR gene polymorphism may influence VD on various physiological processes 22. For example, deleterious mutations in the VDR gene may cause inherited 1,25-(OH)_2_D_3_ resistant rickets, wherein mutated VDR retains the ability to bind to 1,25-(OH)_2_D_3_ but exerts an antagonistic influence on its biological outcomes 23. VDR gene polymorphism (Fok1) may also be associated with calcium regulation 24. VDR can also exhibit nongenomic effects that do not involve VD, which may be related to protein-protein interactions 25.

## VD and cervical cancer

Several ecological studies have investigated the potential impact of UVB exposure on the risk of cervical cancer through the modulation of VD levels. One such study of more than 70,000 cases of black and white patients by Adams et al using the data from the National Cancer Institute found a negative correlation between UV exposure and cervical cancer incidence in America 26. Grant et al. also conducted a multifactorial ecological study in Caucasian Americans from 50 states, which demonstrated a negative association between UVB exposure and cervical cancer mortality 27. Additionally, Chen et al. carried out an ecological study using data from the National Central Cancer Registries (1998-2002), and they reported a 13% decrease in the incidence of cervical cancer for every 10-unit increase in UVB 28, while Grant found a correlation between increased UVB exposure (heat zone and latitude) and reduced mortality of cervical cancer, using data from the National Death Survey in China (1973-1975) 29. Grant's study in twelve administrative areas in France showed a positive relationship between cervical cancer incidence and latitude 30. Taken together, the findings of these ecological studies provide evidence supporting the hypothesis that increased VD levels resulting from UVB exposure may play a role in reducing the risk of cervical cancer (Table 1). However, it is important to note that ecological studies inherently lack the capacity to establish a causal relationship, thus underscoring the necessity for additional research in this regard.

The inhibitory effect of VD on cervical cancer may be related to its potential association with HPV infection. VD has been shown to activate genes and pathways involved in both innate and adaptive immunity, indicating its role in the immune process 18. Previous studies have demonstrated that VD may be utilized as a prophylactic and adjuvant therapy for diseases caused by impaired immune homeostasis 31. Özgü et al. have suggested that VD deficiency may be a risk factor of HPV DNA persistence and related CIN 32. In a cross-sectional survey of 4,343 women, Gupta et al. discovered that serum VD deficiency was associated with a higher risk of HR-HPV infection 33. In a cohort study involving 7,699 female adults Chu et al. found that women with HR-HPV infection had lower serum VD levels 34. Shim et al. observed an increased in HR-HPV infection rates for every 10ng/ml fall in serum 25(OH)D levels in a cross-sectional study of 2353 women in the United States (OR=1.14, 95%CI: 1.02-1.27) 35, while Troja et al. found no correlation between HR-HPV infection rates and the increase of serum 25(OH)D level by 10ng/ml in a narrower age range of 30-50 years old 36. It may be that age range has some influence on the association between VD and HPV infection 35, 36. Nevertheless, these findings have suggested that persistent HPV infection is linked to decreased immune function, and VD may reduce HPV infection rates by improving the body's immunity, thus reducing cervical cancer morbidity and mortality (Table 2).

Numerous epidemiological studies have investigated the relationship between VD and cervical cancer. In a case-control study of 405 cervical cancer patients and 2,025 age-matched controls, Hosono et al. found that Japanese women who consumed higher doses of VD had a lower incidence of invasive cervical cancer, but not CIN3^37^. A randomized, double-blind, placebo-controlled parallel clinical trial involving 58 patients with CIN1 in Iran by Vahedpoor et al. revealed that long-term use of VD supplements for six months was associated with a higher proportion of CIN1 regression than the placebo group 38. Meanwhile, Vahedpoor et al. 39 conducted the similar randomized controlled trial on 58 patients with CIN2/3 and found that the same VD supplement for six months had a positive impact on reducing the recurrence rate in CIN1/2/3. However, when CIN1 was excluded, the difference of recurrence rate between the intervention and placebo groups was no longer statistically significant. The same dose and duration of VD supplementation did not produce the same effect in CIN2/3 patients as in CIN1 patients, possibly due to the greater severity of cervical lesions and more severe symptoms in CIN2/3, which may require a higher dose and longer period of VD supplementation. While these studies have provided a foundation for further investigation, larger and more diverse sample sizes are generally preferred to draw more robust conclusions (Table 3).

According to animal experimentation, the administration of calcitriol alone demonstrated a suppressive effect on cervical tumors in mice. However, there was no evidence indicating that calcitriol enhanced the therapeutic efficacy of radiation treatment 40. There are few animal studies on the effects of VD on cervical cancer, but the current study found that VD may possess anti-carcinogenic properties in the context of cervical cancer, which warrants further investigation to elucidate the underlying mechanisms 40.

## VDR and cervical cancer

The literature on the relationship between cervical cancer and the VDR is limited. A review has reported the role of VDR upregulation in gynecological cancers, with elevated expression levels of VDR in comparison to normal tissues observed in endometrial cancer, ovarian cancer, cervical cancer, and vulvar cancer 9. Contrasting the differences in VDR expression levels in invasive breast cancer tissues, a cohort study has revealed a negative correlation between VDR expression levels and the degree of tumor malignancy. That is to say, as the degree of malignancy worsens, VDR expression levels decrease 41. High VDR expression was inversely associated with the malignancy of prostate cancer and the risk of cancer-related mortality 42. Another cohort study indicated that increased VDR expression was associated with improved overall survival rates, suggesting that VDR levels could serve as a prognostic marker 43. However, Friedrich et al. did not find a significant association between VDR expression levels and cervical cancer pathological staging, differentiation, and lymph node metastasis 44, 45. This disparity might arise from the limited small size of 50 cervical cancer tissues and 15 benign cervical tissues in the study 44. In a review study, combining VDR agonists with standard treatment modalities like aromatase inhibitors has been proposed to enhance the treatment response in breast cancer 46. Therefore, further in-depth investigation is required to elucidate the role of VDR upregulation in cervical cancer progression, prognosis and response to treatment (Table 4).

## Mechanisms underlying the VD-VDR signaling in cervical cancer

While epidemiological studies have demonstrated associations between VD, VDR and a reduced risk of cervical cancer, and certain mechanisms have been investigated, the precise mechanisms by which VD and VDR influencing cervical cancer remain incompletely understood and warrant further exploration.

### Regulation of gene expression by the VD-VDR signaling

Several studies have reported that the VD-VDR signaling in cervical cancer cells can directly impact the expression of key oncogenes and tumor suppressor genes 47, 48. For instance, VDR activation has been shown to downregulate oncogenes like human cervical cancer oncogene-1 (HCCR-1) and upregulate tumor suppressors like p21 and p53, leading to cell cycle arrest and inhibition of cell proliferation 47, 49. Wang et al. conducted research revealing that calcitriol induced cell cycle arrest in the G_1_ phase, thereby inhibiting the proliferation of HeLaS3 cells, through the down regulation of HCCR-1 expression, concomitant with an increase in the expression and promoter activity of p21^47^. Notably, p21 is known to interact with p53, leading to the inhibition of cell proliferation 49. These findings have suggested that VD may play a crucial role in inhibiting the HCCR-1 oncogene of cervical cancer and potentially act as an anti-cervical cancer agent.

Furthermore, the VD-VDR signaling also influences the expression of genes related to immune responses. It can upregulate genes involved in antigen presentation, such as major histocompatibility complex (MHC) class II molecules, enhancing the immune recognition of cancer cells 50, 51. Additionally, VDR can downregulate genes associated with inflammation, thus reducing the pro-inflammatory environment within the tumor microenvironment 52. Previous research also indicated that VDR regulated genes involved in DNA repair pathways. Activation of VDR has been shown to reduce nitrosylation of DNA repair enzymes, thereby maintaining genomic stability 53.

### Regulation of cellular processes by the VD-VDR signaling

In cervical cancer, the VD-VDR signaling has been shown to exhibit inhibitory effects by regulating various cellular processes 47, 54-58, such as cell proliferation, differentiation, and apoptosis. Several studies have investigated associations between VD, VDR and cervical cancer *in vitro* studies 47, 54-58. Calcitriol was found to induce HeLa cells to stop in G_0_/G_1_ phase and inhibited proliferation, by using the cell counting kit-8 assay and flow cytometry 47. Conversely, when employing the ki67 nuclear antigen method and flow cytometry, it was found that cholecalciferol had no effect on SiHa and Caski cells proliferation. Instead, it led to an increased in the sub-G_1_ phase, while no effect was observed on the G_0_/G_1_ phase 54, 55. Furthermore, 25-hydroxycholecalciferol had no effect on SiHa cell proliferation by the ki67 nuclear antigen method. However, it did induce an augmentation in the sub-G1 phase, albeit without instigating cell cycle arrest as elucidated through flow cytometry analysis 57. These findings lead to a plausible hypothesis that the two activation pathways of cholecalciferol *in vivo*, mediated through the liver and kidney, may have specific impacts on cellular behavior. This conjecture is supported by the observed differential effects of various forms of vitamin D on cell proliferation and the cell cycle.

Moreover, the VD-VDR signaling plays an important role in activating both intrinsic and extrinsic apoptosis pathways, which highlights its potential as a therapeutic target for inducing programmed cell death in cervical cancer cells. The VD-VDR signaling has been found to activate the intrinsic apoptosis pathway in cervical cancer cells. This involves the release of pro-apoptotic factors from the mitochondria, such as cytochrome c, which triggers the formation of the apoptosome and subsequently leads to caspase activation and apoptosis 59, 60. In addition to the intrinsic pathway, the VD-VDR signaling can also influence the extrinsic apoptosis pathway. Studies have shown that VDR activation can upregulate death receptors like Fas and Fas ligand (FasL), leading to the activation of caspase and initiation of extrinsic apoptosis 61, 62. While these findings listed above are often based on specific cancer cell lines, it's critical to acknowledge that the response to VDR activation may differ among various cervical cancer subtypes, and extrapolating these results to clinical setting requires caution.

The VD-VDR signaling can also modulate the expression of Bcl-2 family proteins, which are critical regulators of apoptosis. VDR activation has been reported to decrease the expression of anti-apoptotic proteins like Bcl-2 and increase the expression of pro-apoptotic proteins like Bcl-2-associated X protein (Bax), tilting the balance in favor of apoptosis induction 63, 64. However, it's important to note that the interplay between these pathways can be complex and context-dependent, involving multiple factors and feedback loops.

### Epigenetic changes and the VD-VDR signaling

Epigenetic alterations, including DNA methylation, can affect VDR expression and activity. Studies have shown that promoter hypermethylation of the VDR gene can lead to reduced VDR expression in cervical cancer cells. This epigenetic silencing of VDR can impair its tumor-suppressive functions, including the regulation of gene expression and apoptosis 65. Additionally, epigenetic changes can also affect the expression of genes involved in cervical cancer pathogenesis and progression. For example, DNA methylation of tumor suppressor genes can lead to their silencing, promoting uncontrolled cell growth. VDR signaling may play a role in regulating the epigenetic status of some of these genes 63. However, it's essential to recognize that epigenetic modifications are highly context-dependent and can vary among individuals. Moreover, while altered VDR expression due to methylation changes is observed, the downstream effects on gene expression and apoptosis may differ among cervical cancer subtypes.

Epigenetic modifications of histones, such as histone acetylation and methylation, can also influence VDR-mediated effects. Altered histone marks at VDR target gene promoters can impact VDR binding and subsequent gene regulation 66, 67. This highlights the intricate interplay between epigenetic modifications and the VD-VDR signaling in cervical cancer. Nevertheless, the exact mechanisms by which histone modifications interact with VDR signaling in cervical cancer cells remain an active area of research. The specificity and dynamics of these interactions need further elucidation.

### Interactions with other signaling pathways

#### VD and Ether à go-go potassium channels

The Ether à go-go (EAG) family of potassium channels has been implicated in promoting cancer cell proliferation and is expressed in a variety of cancer types 68, 69. EAG1, a member of this family 70, is expressed at low levels in normal tissues but is significantly overexpressed in various cancer types. Inhibition of EAG1 expression has been shown to reduce cell proliferation 71. Notably, EAG1 expression has been detected in cervical cancer tissues and cell lines, with increased expression as the severity of CIN increases 68, 72. Previous research has demonstrated that calcitriol can downregulate the expression of EAG1 mRNA and protein in SiHa cells, leading to reduced proliferation. Moreover, this effect was more pronounced in cells transfected with VDR expression vectors, indicating that VDR played a crucial role in mediating the inhibition of EAG1 gene expression 73. Further studies on SiHa and C33A cells have revealed that calcitriol inhibited EAG1 gene expression at the transcriptional level, with the involvement of VDR 74.

Various studies have reported that estrogen could play a role as a co-factor in the increased risk of cervical cancer in women with HPV DNA^+^^75^. Specially, Díaz et al. discovered that the expression of estrogen receptor-α (ERα) led to a strong upregulation of EAG1 expression in HeLa cells 76, in response to both estradiol and anti-estrogen. Conversely, another study found that estradiol, through G protein-coupled receptor 30 (GPR30), rather than ERα, contributes to the destabilization of genome structure in HPV-infected cells, potentially promoting carcinogenesis 77. This discrepancy may be due to different concentrations of estradiol, resulting in distinct mechanisms of action, which may be explained by the dual effects of estrogen concentrations. Moreover, Dupuis et al. confirmed that estrogen had the capacity to enhance the effect of VD by facilitating the expression of VDR. VD, on the other hand, can down-regulate aromatase expression, reducing the level of estrogen 78. Consequently, as subsequent studies unfold, the determination of the optimal estrogen concentration may have substantial significance for influencing VD in cervical cancer. In previous studies, the HPV oncoproteins E6 and E7 caused the loss of cell protein p53 and retinoblastoma protein pRb^79^. It is noteworthy that EAG1 may be down-regulated by p53 and pRb pathways 76, indicating a potential synergistic effect of estrogen and HPV on EAG1 expression.

The HERG potassium channel, a member of the EAG potassium channel family, has been identified in various cancer types, and HERG mRNA has been detected in HeLa cells 70. Suzuki et al. have observed the expression of the HERG gene in C33A cells, and HERG channel inhibitors have been shown to reduce the G_2_/M phase cell ratio 80. Furthermore, VD has been reported to up-regulate tumor necrosis factor α (TNF-α) in cancer cells 81, leading to an increase in intracellular reactive oxygen species 82. Nevertheless, it is noteworthy that reactive oxygen species may increase the outward current of HERG potassium channels, while its scavengers can reduce the outward current at rest 83. Therefore, it is hypothesized that VD may have a modulatory effect on the HERG potassium channel.

#### Wnt/β-catenin pathway

The abnormal activation of the Wnt/β-catenin signaling pathway is implicated in irregular cell proliferation and differentiation, which can contribute to tumorigenesis. Studies have revealed an elevated rate of abnormal β-catenin protein expression along with the progression of CIN and the development of cervical cancer 84. Notably, HPV E6 and E7 oncoproteins have been shown to up-regulate β-catenin expression, activate the regulated Wnt pathway, and thereby promote cervical cancer progression 85, 86. In related research, VD has exhibited inhibitory effects on the progression of various cancer, such as melanoma 87, Kaposi's sarcoma 88, oral squamous cell carcinoma 89, and ovarian cancer 90, by modulating the Wnt/β-catenin signaling pathway through its interaction with VDR. Therefore, it is reasonable to speculate that VD may inhibit the progression of cervical cancer by binding with VDR, potentially implicating the Wnt/β-catenin signaling pathway as a target for intervention.

#### PI3K-AKT pathway and PI3K-AKT- mTOR pathway

The phosphatidylinositol 3-kinase/Akt (PI3K/AKT) pathway has the capacity to modulate the expression of key oncogenes (e.g. HCCR-1) and tumor suppressor genes (e.g. p53) 91 and elicit the transition between epithelial and stromal tissues 92, thereby serving as a facilitator in the onset and advancement of cervical tumors. While studies have demonstrated VD's ability to impede Non-Small-Cell Lung Cancer progression through the PI3K/AKT pathway 93. This pathway's involvement in cervical cancer remains unexplored. However, the association between this pathway and HCCR-1^94^, which is associated with cervical cancer progression, strengthens the hypothesis that the PI3K/AKT pathway may be instrumental in mediating VD's effects on cervical cancer.

The PI3K-AKT-mTOR pathway is an extension of the PI3K/AKT pathway and includes the mammalian Target of Rapamycin (mTOR) as a key downstream component. The extended pathway is known to regulate essential cellular functions including proliferation, differentiation, and apoptosis 95. In various cancer contexts, VD has been shown to engage with VDR and inhibit tumor progression through the PI3K-AKT-mTOR pathway, as observed in Kaposi's sarcoma cells 96 and non-small cell lung cancer 97. Aberrant activation of the PI3K-AKT-mTOR pathway has been documented in cervical cancer 98.

Notably, VD has demonstrated the ability to impede the growth of HeLa cells by suppressing autophagy and altering mitochondrial homeostasis through modulation of the PI3K-AKT-mTOR pathway 58. However, it is crucial to highlight that studies on the PI3K-AKT-mTOR pathway have primarily focused on HeLa cells and has not encompassed other cervical cancer cell lines, such as Caski, SiHa and C33A cells. Therefore, it remains inconclusive whether the observed effects of VD are attributed to specific characteristics of HeLa cells, such as their HPV typing.

**Figure 2:**
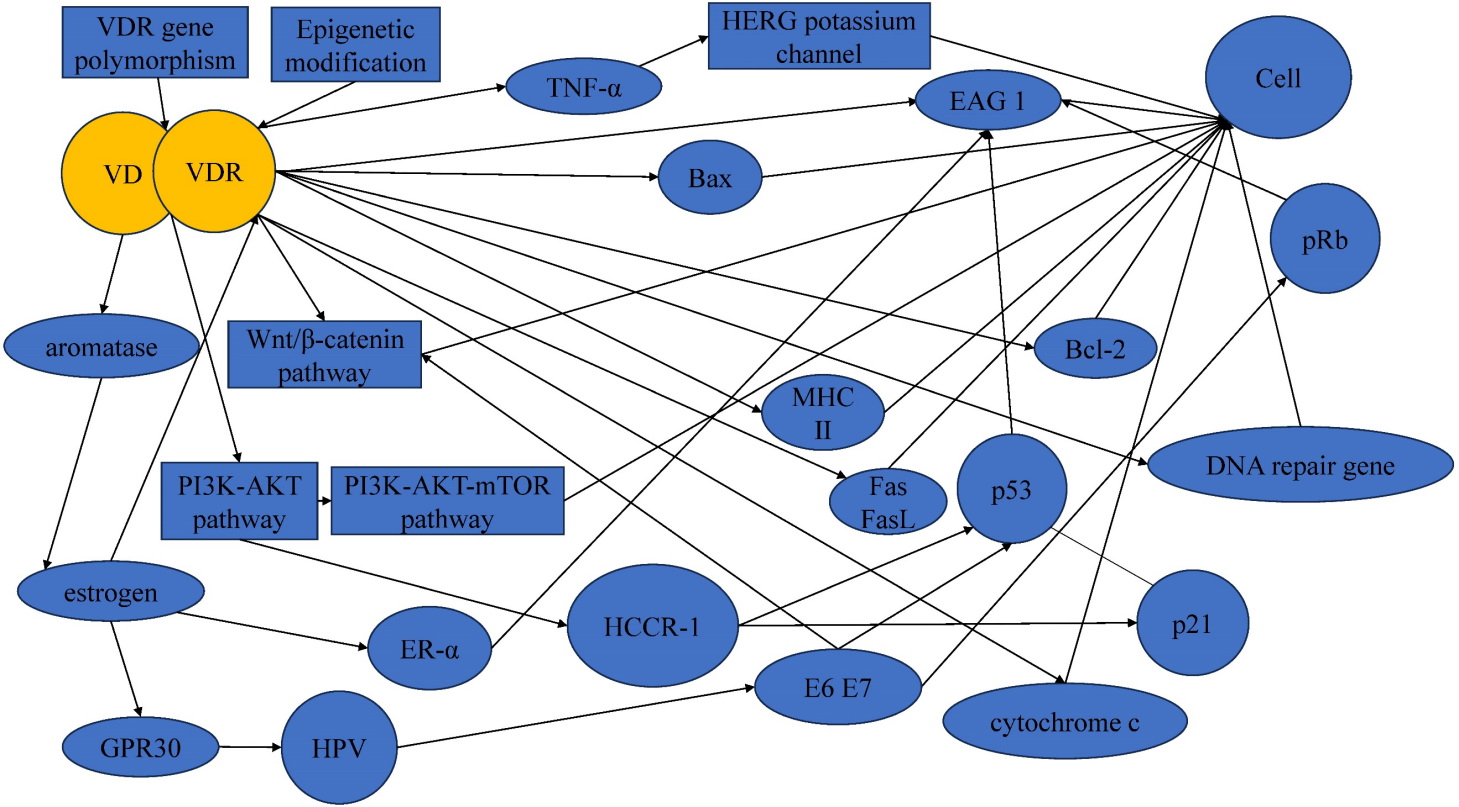
Mechanisms underlying the protective actions of VD in cervical cancer.

It is crucial to acknowledge that the development and progression of cervical cancer involve a complex interplay of multiple factors, and the VD-VDR signaling represents one aspect of this complexity (Figure 2). Thus, the translation of these findings into clinical applications may face challenges related to the specificity, safety, and potential side effects of VDR-targeted therapies.

## VDR gene polymorphism and cervical cancer

The antitumor effect of VDR is influenced by gene polymorphism, which can affect the activity of the VD-VDR complex 99, 100. Certain gene polymorphisms may reduce VDR activity and responsiveness to calcitriol, thereby leading to the progression of cervical cancer. Genetic polymorphic loci such as APa1, Bsm1, Taq1, Fok1, Cdx2 have been extensively studies in the context of tumors 101. Investigations have shown that VDR gene polymorphism is associated with ovarian cancer (Fok1, Apa1), breast cancer (Bsm1, Fok1) and other tumors 102. Phuthong et al. have detected VDR polymorphism (Fok1, Apa1, and Taq1) in 204 patients with cervical squamous cell carcinoma and 204 healthy controls matched by age, and found that Taq1 was associated with cervical cancer in northeastern Thailand, and Taq1 and Fok1 may interact to affect the development of cervical cancer but no association in APa1^103^. Meanwhile, Li et al. found that Fok1 and Taq1 polymorphisms were associated with an increased risk of CIN2+ (CIN2, CIN3 and cervical cancer) within the Shanxi population 104. It is imperative to acknowledge certain limitations in these studies, such as their exclusive focus on cervical squamous cell carcinoma 103, or their restriction to HPV16+ CIN2+ patients, without distinguishing between CIN and cervical cancer patients 104. Notably, these studies did not consider gene-environment interactions, for example, skin color may affect the process of skin synthesis of VD under light, and circulating VD levels may interact with the VDR gene variants to have an impact on cervical cancer risk. Future research should expand the scope of investigation to include other subtypes of cervical cancer, and consider the intricate interplay between genetic and environmental factors. Such endeavors will undoubtedly advance our understanding of the association between VDR gene polymorphism and cervical cancer.

Persistent HPV infection is linked to the impairment of immune function. VDR is expressed in multiple cell types, including immune cells 31, indicating a potential association between VDR and HPV infection. Previous studies have suggested that VDR gene polymorphism is related to viral infection 105, and the polymorphism of the VDR gene may influence the effect of VD by affecting its activity and expression. Nevertheless, to date, empirical examinations that examine the relationship between VDR gene polymorphism and HPV infection remain conspicuously absent from the extant literature, thereby warranting consideration as a prospective avenue of research in forthcoming investigations.

## Interaction of VD with calcium and other vitamins

Although vitamins and minerals are micronutrients, their impact on human physiological processes is profound. Minerals and vitamins, such as calcium (Ca), vitamin A (VA), vitamin B (VB), vitamin C (VC), VD, vitamin E (VE) and vitamin K (VK), have been reported as potential preventive measures against the progression of cervical cancer 9, 106-110. Studies have highlighted the potential significance of VD in impeding cervical cancer progression, warranting further exploration of the interactions of VD with Ca and other vitamins.

VD has been recognized for its traditional role in the regulation of calcium and bone metabolism. VD may collaborate with calcium by regulating calcium metabolism in the body. Exploring the synergistic effects of Ca and VD in cervical cancer holds therapeutic promise, particularly considering the significant role of Ca signaling channels in cervical cancer 111.

VA plays an anti-cancer role through its antioxidant capacity 112. Retinoic acid, a metabolite of VA in the body, exerts its function through the retinoic acid receptor (RAR)-RXR heterodimer, formed by RXR and the nuclear receptor of the RAR family. This complex interacts with retinoic acid response elements (RAREs) within the promoters of retinoic acid-responsive genes 113. Given that both VA and VD interact with RXR, the possibility of antagonistic effects arises. Epidemiological studies have shown that VA levels in both dietary intake (OR=0.59 95%CI 0.49-0.72) and blood (OR=0.60 95%CI 0.41-0.89) are inversely associated with cervical cancer risk 114. Moreover, VA has been shown to inhibit the transcription level of HPV oncogenes 115. Therefore, it is reasonable to speculate that VA can affect EAG1 by affecting HPV oncogenes, but the competitive relationship of RXR also needs to be considered.

Folate, a member of the B vitamin group, mainly uses three folate transporters for cellular uptake: the reduced folate carrier (RFC), the proton-coupled folate transporter (PCFT) and folate receptors (FOLRs). Studies have suggested that VD_3_ and its receptors can increase the expression of PCFT, thus increasing folate intake 116. However, conflicting findings exist, with some studies reporting no effect of VD on folate levels in the body 117. The effect of VD on folic acid metabolism is still controversial, potentially involving undiscovered mechanisms warranting further investigation. Notably, folate receptor ɑ (FRɑ), one of the FOLRs, displays increased expression with the progression of cervical lesions and is highly expressed in cervical cancer 118, 119. FRɑ exerts its influence on p53 and p21 through the ERK1/2 signaling pathway 118. Given that VD can also influence p53 and p21 through various mechanisms, it is reasonable to postulate potential interactions between folate and VD in the context of cervical cancer, although over-supplementation of folate may have a positive effect on precancerous changes 120.

VC exerts its anti-cancer effects by acting on redox imbalances, epigenetic reprogramming an oxygen-sensing regulation of cancer cells 121. Patients with cervical cancer have been reported to exhibit lower levels of VC in both blood levels and dietary intake compared to normal controls 122-124. VC can potentially impact cervical cancer through pathways involving TNF-α and p21^125^, which may interact with the anti-cancer pathway of VD.

VE hinders cancer progression by promoting apoptosis and inhibiting cell proliferation and invasion 126. Epidemiological studies have shown that VE levels in both dietary intake (OR=0.68 95%CI:0.49-0.94) and blood (OR=0.52 95%CI:0.40-0.69) are inversely associated with cervical cancer risk 127. VE consists mainly of tocopherols and tocotrienols, with tocopherol notably inhibiting the AKT signaling pathway to exert its anticancer effects 126. Tocotrienol, another component of VE, can inhibit cell proliferation through several pathways, including the PI3K/AKT pathway, the Wnt/β-catenin pathway and TNF-ɑ 128. The potential relationship between VE and the action pathway of VD suggests the possibility of a synergistic role against cervical cancer.

VK can promote apoptosis, induce cell cycle arrest and overcome drug resistance by inhibiting P-glycoprotein, offering potential value when combined with VD in the treatment of cervical cancer 129. A cross-sectional survey of more than 10,000 women in the United States suggests an association between VK intake and HPV infection, although the relationship appears non-linear 130. VK can produce reactive oxygen species, which leads to apoptosis 131, 132. Given that VD also influences reactive oxygen species, the potential synergy in inhibiting cervical cancer progression warrants consideration.

## Conclusions

In summary, VD and VDR emerge as potential pivotal factors in the occurrence and progression of cervical cancer, potentially reducing disease risk. Although epidemiological studies have established associations between VD, VDR and cervical cancer susceptibility, the empirical support for their preventive efficacy remains notably reliant on a paucity of clinical trial studies. Existing studies underscores that the inhibitory effect of VD on cervical cancer may be mediated through various pathways and factors, including but not limited to the EAG potassium channel, HCCR-1, estrogen and its receptor, p53, pRb, TNF-α, the PI3K/Akt pathway, and the Wnt/β-catenin pathway. However, the extant literature in the realm of cervical cancer remains limited, with a conspicuous dearth in investigations exploring the intricate interplay among diverse molecular pathways and entities.

In addition, while the association between VDR gene polymorphisms and cervical cancer has been elucidated to some extent, there remains an empirical void concerning the mechanistic underpinnings of these polymorphic loci changes in the context of HPV infection and VD. Moreover, an avenue of research conspicuously absent pertains to the relationship between genetic polymorphisms, dietary intake of VD and calcium, and their collective influence on cervical cancer.

VD, as a vitamin, has a certain inhibitory effect on cervical cancer, thereby eliciting interest in the potential contributions of other vitamins in the context of this malignancy. This review briefly alludes to the possible effects of other vitamins on cervical cancer, and explores potential synergistic mechanisms between VD and its vitamin counterparts. While VD's traditional role lies in the regulation of calcium metabolism, the intricate interplay between VD, Ca, and other vitamins has received limited attention in the current literature. Many of the proposed mechanistic processes still require confirmation through future studies.

In conclusion, VD presents a promising avenue for novel therapeutic approaches to cervical cancer. Further research endeavors should seek to elucidate the potential synergistic benefits of combining VD with other anticancer medications, thereby advancing our understanding of effective treatment strategies in this context.

## Figures and Tables

**Table 1:** Ecological research on the association between UVB exposure and cervical cancer.

Authors	Year	Sample size	Date source	Results	Reference
Adams et al	2016	71,209 patients	The National Cancer Institute	The age-adjusted cervical cancer incidence per 100,000 individuals was 4.47, 4.36, and 8.27 for the high (>12363.17 J/m^2^), medium (10647.77-12363.17 J/m^2^), and low (<10647.77 J/m^2^) UV exposure groups, respectively (P<0.001).	26
Grant et al	2006	50 states	State-averaged data for Caucasians for the periods 1950-1969 and 1970-1994	A negative association between UVB exposure and age-adjusted cervical cancer mortality (r=-0.46, P=0.005).	27
Chen et al	2010	30 counties	The Chinese National Central Cancer Registries, 1998-2002	Association between UVB and cervical cancer incidence (RR=0.87, 95%CI 0.80-0.95).	28
Grant	2007	39 counties	National Death Survey, 1973-1975 in China	A negative association between heat zone and cervical cancer mortality (β=-0.61, P<0.001), a positive association between latitude and mortality (β=0.69, P<0.001).	29
Grant	2010	21 continental regions of France	The 12 cancer registries and a publication of the Fédération Nationale des Observatoires Régionaux de la Santé, etc	A positive association between latitude and cervical cancer incidence (r=0.60, P=0.004).	30

Abbreviations: RR, risk ratio; CI, confidence interval.

**Table 2:** Epidemiological research on VD deficiency and HPV infection.

Authors	Year	Sample size	Design	Results	Reference
Özgü et al	2016	85 patients	Case-control study	Women with abnormal cervical smear results and positive HPV DNA had lower levels of VD compared to women with negative HPV DNA.	32
Gupta et al	2021	4343 females	Cross-sectional study	VD deficiency was associated with higher HPV infection (low-risk HPV infection: OR=1.41, 95%CI 1.23-1.61; HR-HPV: OR=1.25, 95%CI 1.04-1.49).	33
Chu et al	2021	7699 females	Cohort study	HPV positive women had lower serum VD levels (P<0.01).	34
Shim et al	2016	2353 females	Cross-sectional study	For every 10 ng/mL reduction in serum 25(OH)D levels, the risk of high-risk HPV infection increased (OR=1.14, 95%CI 1.02-1.27).	35
Troja et al	2020	404 females	Cohort study	Serum VD levels were not associated with HR-HPV infection.	36

Abbreviations: OR, odds ratio; CI, confidence interval.

**Table 3:** Epidemiological research on VD and cervical cancer.

Authors	Year	Sample size	Design	Results	Reference
Hosono et al	2010	405 cervical neoplasias and 2025 age-matched non-cancer controls in Japan	Case-control study	Compared with Q1, VD intake had an inverse association in invasive cervical cancer (Q2: OR=1.03, 95%CI 0.74-1.44; Q3: OR=0.80, 96%CI 0.56-1.15; Q4: OR=0.64, 95%CI 0.43-0.94, Ptrend=0.013). However, there were no association between VD intake and CIN3 (Ptrend=0.109).	37
Vahedpoor et al	2017	58 CIN1 patients	Randomized Controlled Trial	CIN1 regression rate of VD group vs. placebo group: 84.6% vs. 53.8% (P=0.01).	38
Vahedpoor et al	2018	58 CIN2/3 patients	Randomized Controlled Trial	Comparing the VD group's CIN1/2/3 recurrence rate to the placebo group: 18.5% vs. 48.1% (P=0.02). However, the changes were not statistically significant (P=0.15) when CIN1 was excluded from the analysis.	39

Abbreviations: Q, quartile; OR, odds ratio; CI, confidence interval.

**Table 4:** Research on VDR and risk of cancer.

Authors	Year	Sample size	Design	Results	Reference
Deuster et al	2017	-	Systematic review	Compared with normal tissues, VDR was upregulated in cancers such as endometrial cancer, ovarian cancer, cervical cancer and vulvar cancer.	9
Huss et al	2019	718 patients	Cohort study	VDR positive expression was associated with favorable tumor characteristics such as smaller tumor size and lower tumor grade. High VDR expression was associated with a reduced risk of breast cancer death.	41
Hendrickson et al	2011	841 patients	Cohort study	Men with the highest VDR expression had a significantly reduced risk of fatal prostate cancer compared to the lowest quartile (HR=0.17; 95% CI, 0.07- 0.41).	42
Ditsch et al	2012	82 patients	Cohort study	Patients with higher VDR expression levels had higher overall survival.	43
Friedrich et al	2002	50 cervical cancer tissues and 15 Benign cervical tissues	Case-control study	There were no significant correlations between the expression of VDR and tumor stage, histological type, proliferation marker KI-67 and tumor suppressor gene p53 expression.	44
Friedrich et al	2003	-	Case-control study	The expression level of VDR was higher in cervical cancer tissues compared with normal tissues. However, upregulation of VDR was not associated with cell proliferation. There was no statistical association between VDR expression and histological type or grade.	45
Krishnan et al	2012	-	Systematic review	Calcitriol reduced the expression of aromatase, and the combination of calcitriol and aromatase inhibitors may have a better anti-cancer effect.	46

Abbreviations: HR, hazard ratio; CI, confidence interval.

## Abbreviations

CIN: cervical intraepithelial neoplasia
HR-HPV: high-risk human papillomavirus
HPV: human papillomavirus
VD: vitamin D
VDR: vitamin D receptor
UVB: ultraviolet B
RXR: retinoid X receptor
RR: risk ratio
CI: confidence interval
OR: odds ratio
Q: quartile
HR: hazard ratio
HCCR-1: human cervical cancer oncogene-1
MHC: major histocompatibility complex
FasL: Fas ligand
Bax: Bcl-2-associated X protein
EAG: Ether à go-go
Erα: estrogen receptor-α
GPR30: G protein-coupled receptor 30
TNF-α: tumor necrosis factor α
PI3K/AKT: phosphatidylinositol 3-kinase /Akt
mTOR: mammalian target of the rapamycin
Ca: calcium
VA: vitamin A
VB: vitamin B
VC: vitamin C
VE: vitamin E
VK: vitamin K
RAR: retinoic acid receptor
RAREs: retinoic acid response elements
RFC: reduced folate carrier
PCFT: proton-coupled folate transporter
FOLRs: folate receptors
FRɑ: Folate receptor α
